# Supplementary material for: Development and Internal Validation of a Model for Predicting Overall Survival in Subjects with MAFLD: A Cohort Study
Source: J Clin Med. 2024 Feb 19;13(4):1181. doi: 10.3390/jcm13041181 (PMC10889818; doi:10.3390/jcm13041181)
Supplement: Supplementary file 1 [file jcm-13-01181-s001.zip › Supplementary materials.pdf]

Development and Internal Validation of a Model for Predicting Overall Survival in Subjects with MAFLD: A Cohort Study

Table S1. Test of proportional-hazards assumption on the basis of Schoenfeld residuals after fitting Cox model .

	rho	Chi^2	*p-value*
Age at enrollment (years)	-0.04	0.89	0.3448
Sex	-0.08	1.76	0.1849
Widowhood	0.07	1.23	0.2669
SBP	0.02	0.08	0.7836
DBP	0.05	0.69	0.4059
GGT (µkat/L)	0.02	0.09	0.7669
TC (mmol/L)	-0.06	1.00	0.3168
Glucose	0.10	2.68	0.1014
ALP (µkat/L)	-0.004	0.01	0.9410
Global test		11.08	0.2705

GGT: γ-Glutamyl transferase; SBP: Systolic Blood Pressure; DBP: Diastolic Blood Pressure; TC: Total Cholesterol; ALP: Alkaline Phosphatase.

Table S2. Cox Survival Models: Effect of Removing Variables on Harrell'C and Explained Variation (R^2_D)

Variable removed	R^2_D	Harrell'C	Order
--	0.6845	0.8422	
Age at enrollment (yrs)	0.4952	0.7476	1
GGT (µkat/L)	0.6786	0.8393	2
SBP	0.6800	0.8400	3
Glucose (mmol/L)	0.6802	0.8401	4
Sex	0.6819	0.8409	5
DBP	0.6825	0.8413	6
TC (mmol/L)	0.6827	0.8414	7
ALP	0.6830	0.8415	8
Widowhood	0.6836	0.8418	9

GGT: γ-Glutamyl transferase; SBP: Systolic Blood Pressure; DBP: Diastolic Blood Pressure; TC: Total Cholesterol; ALP: Alkaline Phosphatase.

Table S3. Baseline characteristics of the Development Cohort by age classes

	Age classes (years) at enrollment				
	<40	40-54	55-69	≥70	*p-value*[d]
N[a]	241 (16.0)	553 (36.7)	619 (41.1)	93 (6.2)	
Sex[a]					
Female	46 (19.1)	140 (25.3)	248 (40.1)	35 (37.6)	<0.001
Male	195 (80.9)	413 (74.7)	371 (59.9)	58 (62.4)	
BMI [b] (kg/m2)	30.19 (4.87)	30,60 (4,93)	31,41 (5,07)	31,32 (4,67)	0.003
Glucose [b] (mmol/L)	105.95 (18.56)	109,63 (20,25)	118,87 (37,65)	126,54 (36,63)	<0.001
TC [b] (mmol/L)	199.17 (40.34)	209,30 (39.45)	206.18 (40.59)	181.67 (36.35)	<0.001
GGT [b] (μkat/L)	16.96 (11.67)	19.05 (15.05)	20.25 (19.70)	23.75 (28.11)	0.008
Observation time[c] (years)	15.85 (15.74-15.95)	15.89 (15.63-16.13)	16.09 (15.17-16.46)	7.17 (4.13-10.48)	<0.001
Age at death [b] (years)	51.4 (3.16)	63.6 (4.78)	76.5 (4.64)	82.0 (3.24)	<0.001
Status[a]					
Alive and/or Censored	235 (97.5)	517 (93.5)	480 (77.5)	3 (3.2%)	<0.001
Dead	6 (2.5)	36 (6.5)	139 (22.5)	90 (96.8)	
Widower [a]					
No	239 (99.2)	542 (98.0)	575 (92.9)	69 (74.2)	<0.001
Yes	2 (0.8)	11 (2.0)	44 (7.1)	24 (25.8)	
Subtype 1[a]					
No	22 (9.1)	47 (8.5)	33 (5.3)	9 (9.7)	0.085
Yes	219 (90.9)	506 (91.5)	586 (94.7)	84 (90.3)	
Subtype 2[a]					
No	229 (95.0)	521 (94.2)	601 (97.1)	84 (90.3)	0.011
Yes	12 (5.0)	32 (5.8)	18 (2.9)	9 (9.7)	
Subtype 3[a]					
No	233 (96.7)	521 (94.2)	522 (84.3)	70 (75.3)	<0.001
Yes	8 (3.3)	32 (5.8)	97 (15.7)	23 (24.7)	

rMED score[a]					
Low	93 (40.1)	183 (34.3)	112 (18.5)	21 (22.6)	<0.001
Medium	120 (51.7)	271 (50.7)	343 (56.6)	55 (59.1)	
High	19 (8.2)	80 (15.0)	151 (24.9)	17 (18.3)	
Olive Oil consumption [b] (gr/die)	25.10 (14.43)	28.49 (15.32)	33.61 (19.48)	40.41 (21.75)	<0.001
Wine consumption [b] (ml/die)	117.64 (173.92)	167.21 (213.85)	175.31 (218.60)	171.99 (206.96)	0.004
Beer consumption [b] (ml/die)	55.56 (121.51)	63.58 (143.60)	51.98 (130.63)	24.03 (79.00)	0.052
Liquor consumption [b] (ml/die)	19.19 (78.45)	31.84 (114.12)	22.61 (89.22)	5.18 (18.89)	0.047
Fruit consumption spring-summer [b](gr/die)	215.10 (221.36)	228.33 (259.50)	198.41 (220.95)	208.07 (224.21)	0.20
Fruit consumption autumn-winter [b] (gr/die)	256.43 (266.15)	324.11 (330.22)	313.60 (292.94)	374.42 (365.12)	0.007
Vegetable consumption [b] (gr/die)	219.35 (173.65)	212.60 (197.10)	238.72 (228.09)	254.94 (225.92)	0.094
Legume consumption [b] (gr/die)	37.23 (29.35)	41.93 (39.32)	44.28 (36.66)	37.26 (33.73)	0.048
Fish consumption [b](gr/die)	33.32 (24.11)	37.16 (31.15)	37.82 (43.35)	33.52 (31.55)	0.33
Dairy products consumption [b](gr/die)	32.81 (27.95)	31.58 (29.51)	25.98 (34.76)	26.67 (28.67)	0.005
Kcal days [b]	2424.75 (874.91)	2382.85 (886.62)	2100.11 (939.19)	2038.82 (885.77)	<0.001

Fruit consumption spring-summer: Peaches, Watermelon, Apricots, Cherries, Grapes, White Melon. Fruit consumption autumn-winter: Apples, Pears, Citrus Fruits And Kiwis. Dairy products: Mozzarella, Ricotta and Provolone. Vegetables: Aubergines, Courgettes, Spinach, Cabbage, Cauliflower, Turnip Greens, Cucumbers, Watermelons, Green Beans, Fava Beans with Vegetables, Carrots, Artichokes, Peppers, Fennel, Celery, Swiss Chard, Chicory, Green Salad. Salad Tomatoes. Legumes: Broad Beans, Chickpeas, Beans, Lentils and Peas. Fish: Octopus, Cuttlefish, Squid, Shrimps, Anchovies, Sardines, Mussels, Mullet, Mackerel.

rMed: relative Mediterranean score; BMI: Body Mass Index; TC: Total Cholesterol; GGT: γ-Glutamyl transferase; Subtype 1: Hepatic Steatosis and Overweight/Obesity; Subtype 2: Hepatic steatosis and the presence of at least two metabolic abnormalities; Subtype 3: Hepatic steatosis and type 2 diabetes mellitus. Cells showing subject's characteristics contain [a]Number. (Percentage) Percentages calculated for the column; [b]Mean±(SD); [c]Median (IQR). [d]ANOVA for continuous and Chi2 for categorical variables were applied where appropriate.
