# Supplementary figures and images for: Development and Internal Validation of a Model for Predicting Overall Survival in Subjects with MAFLD: A Cohort Study
Source: J Clin Med. 2024 Feb 19;13(4):1181. doi: 10.3390/jcm13041181 (PMC10889818; doi:10.3390/jcm13041181)

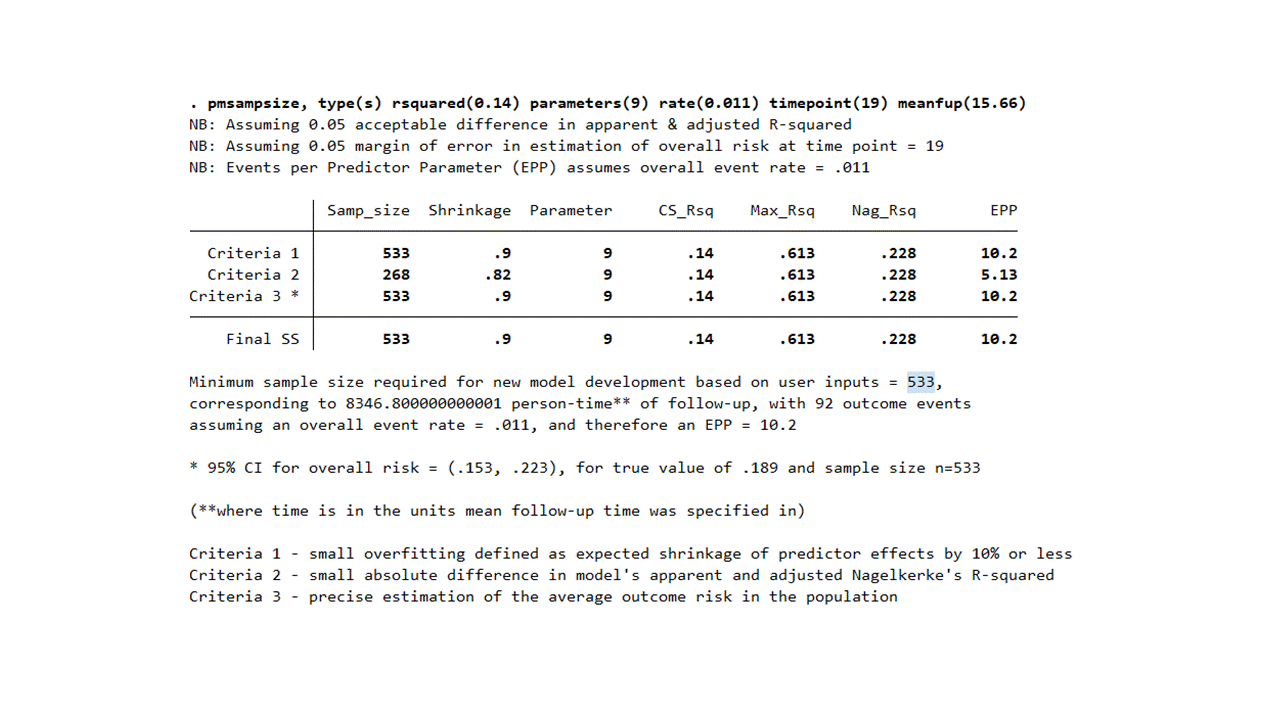

Supplement: Supplementary file 1 [file jcm-13-01181-s001.zip › Figure S1.gif]

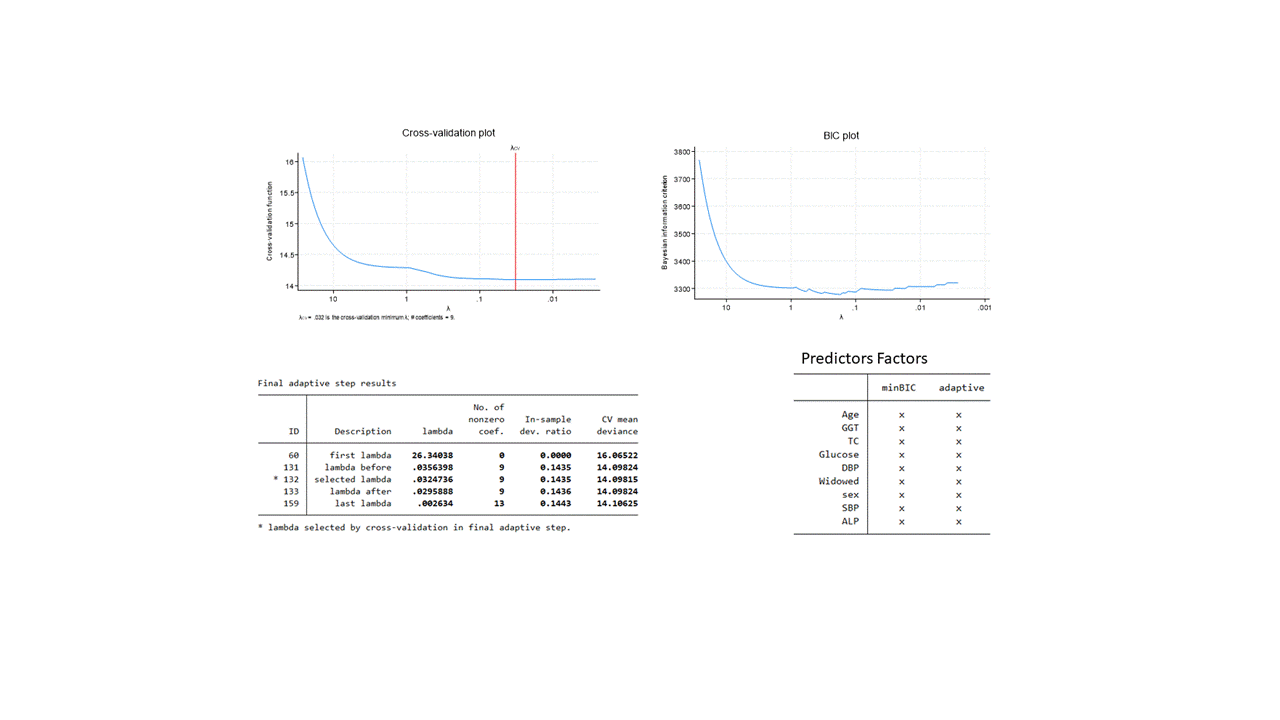

Supplement: Supplementary file 1 [file jcm-13-01181-s001.zip › FigureS2.gif]
